# Attitudes and knowledge of nurses working at night and sleep promotion in nursing home residents: multicenter cross-sectional survey

**DOI:** 10.1186/s12877-023-03928-9

**Published:** 2023-03-31

**Authors:** Denise Wilfling, Almuth Berg, Jonas Dörner, Natascha Bartmann, Thomas Klatt, Gabriele Meyer, Margareta Halek, Ralph Möhler, Sascha Köpke, Martin N. Dichter

**Affiliations:** 1 Institute of Social Medicine and Epidemiology, Nursing Research Unit, University of Lübeck, Lübeck, Germany; 2 Institute for Health and Nursing Science, Medical Faculty, Martin Luther University Halle- Wittenberg, Halle (Saale), Germany; 3 German Center of Neurodegenerative Diseases (DZNE), Witten, Germany; 4 School of Nursing Science, Faculty of Health, Witten/Herdecke University, Witten, Germany; 5 Department of Nursing Science, Faculty of Health, Witten/Herdecke University, Witten, Germany; 6 Institute for Health Services Research and Health Economics, Centre for Health and Society, Medical Faculty and University Hospital Düsseldorf, Heinrich-Heine-University Düsseldorf, Düsseldorf, Germany; 7 Institute of Nursing Science, University of Cologne, Faculty of Medicine and University Hospital Cologne, Cologne, Germany

**Keywords:** Sleep, Sleep promotion, Educational needs, Nurses’ attitudes, Nursing homes, Germany

## Abstract

**Background:**

Sleep disturbances are common in nursing home residents and challenging for their nurses. Knowledge about sleep and sleep promoting factors is essential to provide adequate sleep management, where nurses play a key role. Therefore, nurses’ knowledge and attitudes towards sleep and sleep promoting interventions is important as enabling or inhibiting factor for successful sleep management.

**Methods:**

A multicenter cross-sectional study was conducted among nurses working wholly or partially at night in nursing homes in Germany. Data were collected between February and April 2021 via online or paper and pencil questionnaires, comprising 56 items. Nursing homes were recruited through existing cooperation with the study centers as well as via nursing home registers.

**Results:**

Finally, 138 nursing homes participated and 271 nurses completed the survey. Nurses agreed that sleep disturbances are an important topic with important impact on resident’ health. Although, the assessment of sleep was seen as nurses’ responsibility, only 40 nurses (14.7%) stated that residents’ sleep was always documented. Only 21.7% reported the availability of policy documents providing guidance regarding the management of sleep disturbances. The vast majority (93.2%) reported never having received training about sleep and management of sleep disturbances after their basic nursing training.

**Conclusions:**

Our results indicate that nurses working at night can play an important role in residents’ sleep promotion. The findings indicate nurses’ educational needs regarding sleep and sleep promotion. Nursing homes should implement institutional guidelines in order to promote residents’ sleep based on adequate evidence-based non-pharmacological interventions.

**Supplementary Information:**

The online version contains supplementary material available at 10.1186/s12877-023-03928-9.

## Background

Restful and healthy sleep is essential for health and wellbeing, and necessary for physical and psychological functioning [1, 2]. Sleep basically consists of two stages: rapid eye movement (REM) and non-REM (NREM) sleep. NREM sleep consists of three different sub-stages (N1-N3). N1 is the lightest stage of sleep, while N2 is characterized by deeper sleep comprising most of the sleep time. N3 is defined as the deepest NREM sleep stage in which it is difficult to wake up. REM sleep is the phase associated with dreaming with little body movement, while the brain is active similar to wakefulness. Therefore, REM sleep is not considered as the restful phase of sleep [3].

With increasing age, REM sleep is often reduced, while the first stage of light NREM sleep is increased. Therefore, older people tend to sleep less and less deeply [1, 4]. Sleep disturbances in older people include difficulty falling asleep, frequent nocturnal awakenings, early morning awakenings and daytime sleepiness [5].

Sleep disturbances are very common in nursing home residents, challenging their nurses [6]. One epidemiological study reported that up to 50% of older people are suffering from at least one symptom of sleep disturbances [7]. Previous studies reported associations between poor sleep of older people with a variety of health outcomes such as cardiovascular diseases [8, 9], pain [10], depression and anxiety [10], cognitive impairment [11] and physical disability [12].

There are a number of factors contributing to sleep disturbances in nursing home residents, such as environmental (e.g. light exposure, nocturnal care, room sharing) and behavioral factors (e.g. reduced physical and social activities, daytime napping) [6, 13]. These challenges are exacerbated by the fact that mostly people with cognitive impairments live in nursing homes, who are especially affected by sleep disturbances [14]. The increasing number of nursing home residents with sleep disturbances contribute to nurses’ increased distress and burden [15].

Since it is known that pharmacological interventions are not effective to treat sleep disturbances for a longer period of time [16], non-pharmacological interventions have been recommended [17–20], but the implementation of these interventions remains challenging [21, 22]. The management of sleep problems includes daytime as well as nighttime interventions [23]. Since nurses play a key role in the implementation of these interventions, nurses’ knowledge and attitudes towards sleep and sleep promotion may be an important enabling or inhibiting factor for successful sleep management [24]. So far, little is known about the attitudes and knowledge of nurses regularly working in night shifts. We therefore explored attitudes and knowledge of nurses working at night towards sleep and sleep promotion in order to develop and implement evidence-based non-pharmacological interventions as we actually do in the MoNoPol-Sleep Study [25].

## Methods

### Study design

A multicenter cross-sectional study in nursing homes in Germany was conducted. Data collection took place between February and April 2021 via online or paper and pencil questionnaires. The study was approved by the ethics committee of the German Society of Nursing Science (no. 20–016).

### Recruitment and sample

Recruitment was carried out by three study centers: Martin Luther University Halle-Wittenberg (Saxony-Anhalt), University of Lübeck (Schleswig–Holstein) and German Center for Neurodegenerative Diseases (DZNE) Witten (North Rhine Westphalia). For this survey, the recruitment of nursing homes was carried out on the basis of nursing home registers of the federal states of Germany (Saxony-Anhalt, Schleswig–Holstein and North-Rhine Westphalia) and established cooperation’s between study centers and participating nursing homes due to other research projects. Additionally, nursing homes participating in the MoNoPol-Sleep Study [25] were included. Institutions were invited either via e-mail or telephone. Since only a small number responded to the written invitation via e-mail, all nursing homes were contacted again via telephone after two weeks. Reasons for non-participation of nursing homes were not documented. Head nurses or nursing home managers were informed about the study and the inclusion criteria of night nurses (fully licensed nurses or nursing assistants). Head nurse or the nursing home managers identified and contacted night nurses meeting the inclusion criteria and asked them to participate in the survey if they were interested in the survey topic. Therefore, there is no information available how many nurses were eligible but did not participate.

Nurses were eligible for the survey if they were regularly working in night shifts. For this study we defined that nurses are regularly working in night shifts if they had worked at least three night shifts within the last three months prior to data collection. Typically, the length of a night shift is between 8 to 10 h depending on the nursing home. Nurses had to be on a working contract of at least 50% of the regular working hours and had to give informed consent to participate in the study. Based on the German conditions night nurses were defined as nurses with a three year education as well as nursing assistants with at least one year of nursing assistant training. In Germany, the scope of practice differs between nurses and nursing assistants. For example, the planning of the nursing process is the exclusive responsibility of nurses. However, during night shifts in nursing home both level of qualification often performs the same tasks, with a few exceptions (e.g., administering medication), which is why we included both groups.

### Procedure

The survey was accessible either online or through paper and pencil, depending on participants’ preferences. All data were collected anonymous on the level of nurses, using pseudonymization on the level of nursing homes.

To ensure standardized data collection, head nurses or nursing home managers were introduced to the data collection procedure and the standardized questionnaire, and subsequently informed nurses fulfilling the inclusion criteria. Nurses participating in the paper and pencil survey received information about aim and content of the study on the first page of the questionnaire. Furthermore, they received information that consent was provided by filling in the questionnaire. Paper and pencil questionnaires were returned with prepaid envelops.

The online survey was carried out with LimeSurvey software [26]. The procedure of the online survey followed the recommendations for improving web surveys [27]. If nursing homes participated in the online survey, head nurses received the survey link via e-mail including an individual access code. They forwarded this email to the nurses meeting the inclusion criteria. The nursing home-specific link led the nurses directly to the survey after entering the access code. Before the survey started, nurses received information about aim and content of the study. Furthermore, they were informed that consent was provided by filling in the questionnaire. After submitting the questionnaire, no withdrawal of participation was possible anymore. However, while the questionnaire was being filled out, there was always the possibility to leave the survey. In that case, no data were recorded. Based on recommendations to increase response, for a reminder e-mail to the head nurse was sent one week after the initial e-mail. After another week, a second reminder was sent. Potential participants had a total of three weeks to take part in the survey [28, 29].

### Measurements

We developed a questionnaire comprising 56 items, including items from available questionnaires [30–33] and self-developed items, used in an earlier survey [15]. Topics covered by the questionnaire are: (i) Attitudes towards sleep and sleep disturbances, (ii) Importance of sleep in nursing homes, (iii) Knowledge about sleep and sleep promotion, (iv) Education and training about sleep and sleep promotion, (v) Attitudes towards pharmacological interventions to promote sleep, and (vi) Sociodemographic variables. More details about the questionnaire are presented in Table S1 (see supplementary file).

The draft version was piloted in a two-step approach: First, feasibility testing was carried out by presenting the questionnaire to five health or nursing scientists for critical assessment. After their feedback, the questionnaire was modified. In a further step the online survey was drafted and piloted by ten nurses who reviewed comprehensibility and feasibility of the online questionnaire. Based on their feedback, final adaptions of the layout were made. After completion of the online questionnaire, a corresponding paper and pencil version was created.

### Data analysis

Data from the paper and pencil survey were entered into SPSS v. 22 [34]. Plausibility checks were performed during data entry. After data entry, one researcher conducted a first validity check by reviewing the logical connection between data. Implausibility checks were performed by checking if all fields which should contain data were really filled. In the case of implausibly, data were checked and corrected or deleted. To further ensure data quality, all data were checked by a second researcher. Data from the online survey were exported into an Excel file and imported to SPSS. Again, plausibility checks were performed. Data were analyzed descriptively, presenting means and standard deviation. Frequency distribution was calculated for categorical data. Descriptive analyses were performed using the software SPSS v. 22 [34]. Open-ended items referring to knowledge and incident reports were categorized into themes by one researcher and checked by a second person.

## Results

A total of 183 (out of 773) nursing home head nurses or nursing home managers agreed to support the study (*n* = 108 in the paper and pencil survey; *n* = 75 in the online survey). Finally, 138 nursing homes with 271 nurses participated in the survey. Participants’ characteristics are displayed in Table 1.

**Table 1:** Sample characteristics (*n* = 271)

Age, years, number (%)	
< 20	1 (0.4)
20—30	44 (18.6)
31—40	74 (31.4)
41—50	50 (21.2)
> 50	67 (28.4)
Women, number (%)	161 (76.3)
Contract hours, number (%)	
Full time	123 (59.7)
Part time	83 (40.3)
Years working in elderly care, mean (SD)	16.8 (± 9.2)
Level of healthcare training, number (%)	
Fully licensed nurses	192 (80.3)
Nurse assistant	47 (19.7)
Special training*, number (%)	
Yes	101 (45.5)
No	121 (54.5)

^*^E.g. in nursing management, palliative care, gerontopsychiatric care, wound management

Missing values were pairwise excluded

The number of participating nurses working at night per nursing home varied between one and fourteen (median 1). The majority of nursing homes worked with permanent night staff and frequently with temporary employed agency nurses, who could not be contacted by head nurses. Therefore, frequently only one or two nurses per nursing home met the inclusion criteria. We received 27 paper and pencil questionnaires without an ID, which therefore those could not be assigned to any nursing home. Since data was not analyzed on the level of nursing homes, questionnaires without an ID were also included in data analysis. The study flow is visualized in Figure S1 (see supplementary file).

### Attitudes towards sleep and sleep disturbances

**Fig. 1:**
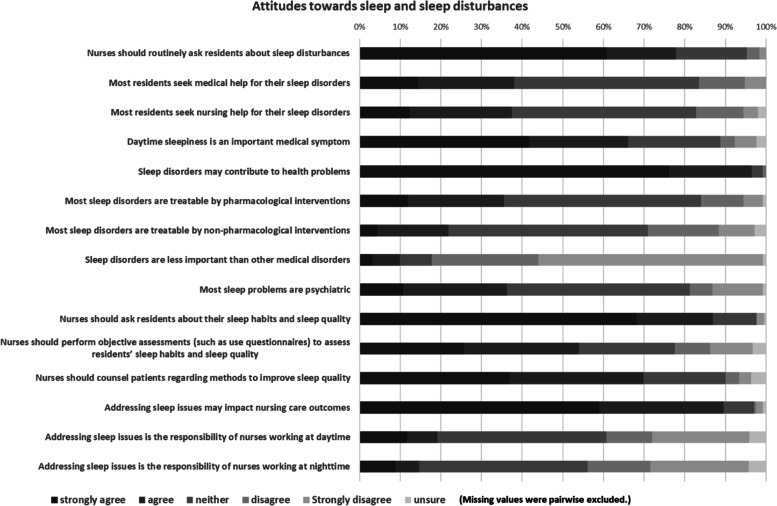
Descriptive results regarding attitudes towards sleep and sleep disturbances

Overall, nurses agreed that sleep disturbances may contribute to health problems (96.4%, *n* = 192/252 strongly agreed, *n* = 51/252 agreed) and may impact nursing outcomes (i.e. outcomes as result of nursing interventions) (89.6%, *n* = 142/241 strongly agreed, *n* = 74/241 agreed). However, 53.9% (*n* = 62/241 strongly agreed, *n *= 68/241 agreed) stated that nurses should perform standardized assessments to evaluate residents’ sleep habits and sleep quality. Responses to questions regarding attitudes towards sleep and sleep disturbances are presented in Fig. 1. Numbers and percentages for each item are summarized in Table S2 (see supplementary file).

### Importance of sleep in the nursing home

**Fig. 2:**
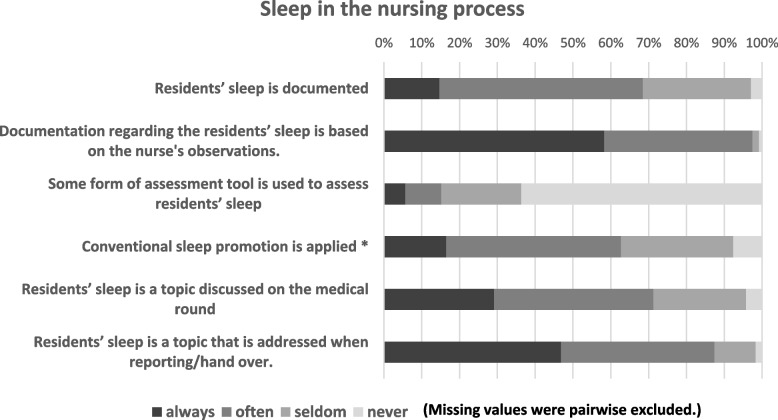
Descriptive results regarding the importance of sleep in nursing homes

Few nurses (14.7%, *n* = 35/238) stated that residents’ sleep was always documented. Documentation of sleep was mainly based on nurses’ observations and 64.3% (*n* = 148/230) stated not to use an assessment tool. Responses to questions regarding sleep within the nursing communication and documentation are presented in Fig. 2. Numbers and percentages for each item are summarized in Table S3 (see supplementary file).

**Fig. 3:**
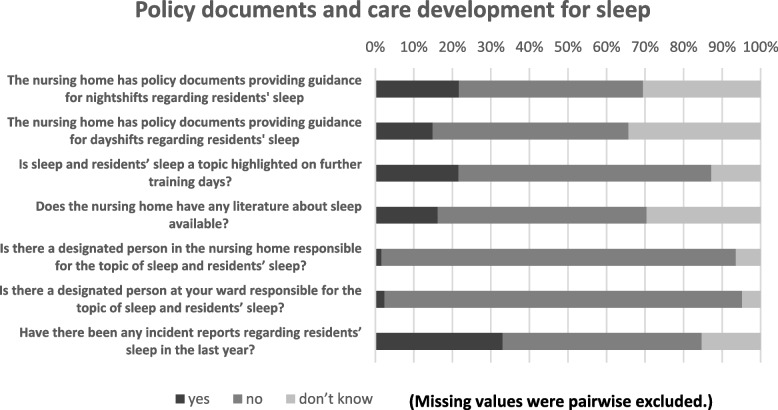
Descriptive results regarding policy documents and care planning for sleep

Concerning guidelines for the management of residents’ sleep disturbances and further training (Fig. 3 and Table S4), only 21.7% (*n* = 54/249) reported that the nursing home has policy documents providing guidance for night shifts on residents’ sleep and only 14.9% (*n* = 37/248) stated the nursing home has policy documents for day shifts. Two thirds (65.6%, *n* = 164/250) of the participants stated that sleep had not been addressed in further training. There are frequent incident reports related to sleep disturbances (33.1%, *n* = 82/248), with most frequently reported incidents being accidental falls (*n* = 47), wandering (*n* = 30), restlessness (*n* = 14), endangering others and oneself (*n* = 5), aggressive behavior (*n* = 4) and screaming (*n* = 3). Numbers and percentages for each item are summarized in Table S4 (see supplementary file). Additionally, on a numeric rating scale from zero to ten (0 = not important; 10 = very important), nurses (*n* = 252) stated that sleep is an important topic in nursing homes (mean 7.47; SD ± 2.3, range 0–10).

### Knowledge about sleep and sleep promotion

The following results are based on five open ended questions summarized in Table S5 (see supplementary file). Since not every participant answered the questions, the n is given here for each question. The participating nurses working at night (*n* = 242) mentioned a disturbed day-night-rhythm (*n* = 83), dementia (*n* = 59), a lack of social and physical activities (*n* = 59), restlessness (*n* = 59), pain (*n* = 29), side effects of medication or inappropriate medication (*n* = 24), and sleepiness during daytime (*n* = 21) as main reasons for sleep disturbances. The most often mentioned (*n* = 233) interventions to promote sleep were daytime activities (*n* = 67), sleep rituals (*n* = 46), ensuring a quiet environment (*n* = 37), and structured day-to-day routines (*n* = 32). For nurses (*n* = 199), the most important competencies needed to promote sleep were empathy (*n* = 46), observation of residents in order to recognize reasons for sleep disturbances (*n* = 18), patience (*n* = 14), and expertise (*n* = 14). From *n* = 172 nurses *n* = 77) stated that non-pharmacological interventions improved residents’ sleep. Twenty nurses reported that they did not have any positive experiences with non-pharmacological interventions to avoid sleep disturbances. Also twenty nurses stated never made negative experiences with non-pharmacological interventions, while other nurses complained that non-pharmacological interventions were not effective and residents showed an increased risk for accidental falls (*n* = 25) and increased restlessness (*n* = 24). Six nurses regarded non-pharmacological interventions as very time consuming.

On a numeric rating scale from 0 (no knowledge) to 10 (perfect knowledge), nurses (*n* = 257) reported a mean knowledge of 5.69 (SD ± 2.1, range 1–10).

### Education and training about sleep and sleep promotion

The majority of the participants (93.2%, *n* = 232/249) reported to never have received training about sleep and management of sleep disturbances after their basic nursing training.

Most of nurses obtained knowledge from self-study (63.7%, *n* = 163/256), or from the experiences with a relative suffering from sleep disturbances (37.1%, *n* = 95/256). Only 3.1% (*n* = 8/2656) reported that their knowledge is related to training by a sleep experts.

### Attitudes towards pharmacological interventions to promote sleep

**Fig. 4:**
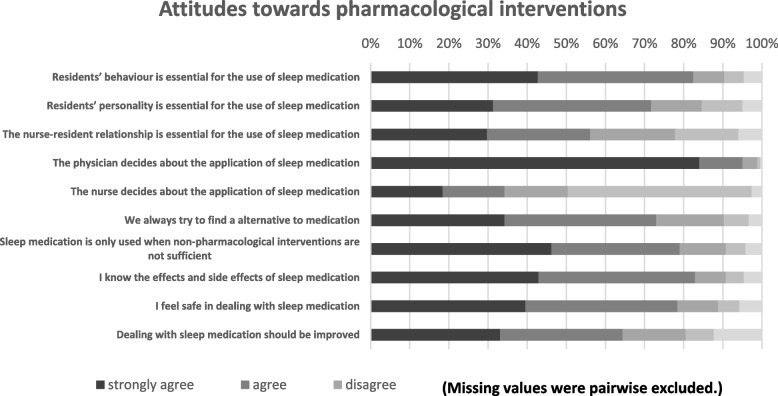
Descriptive results regarding attitudes towards pharmacological interventions to promote sleep

The majority see the prescription as physicians’ responsibility (95.0%; *n* = 199/237 strongly agreed, *n* = 26/237 agreed). Frequently, participants reported attempts to find alternatives to sleep medication (73%, *n* = 81/273 strongly agreed, *n* = 92/273 agreed), but if non-pharmacological interventions are not sufficient, a need for sleep medication is seen (79%, *n* = 110/238 strongly agreed, *n* = 78/238 agreed). Responses to questions regarding attitudes towards pharmacological interventions to promote sleep are presented in Fig. 4. Numbers and percentages for each item are summarized in Table S6 (see supplementary file).

## Discussion

The results of this survey show that nurses recognize residents’ sleep as an important topic and the assessment of sleep as their professional responsibility. Based on the lack of studies addressing night nurses working in nursing homes, the following discussion reflects the results in comparison to findings from other studies concerning the attitudes of health professions in different health care settings.

Nurses in this study stated that sleep disturbances impact health problems and nursing outcomes. Gellerstedt et al. (2019) found similar results with 94.3% of nurses believing that sleep is a nursing topic and 86.8% reporting that sleep disturbances have negative effects on patients’ recovery. Likewise, physiotherapists expressed their views in a study by Siengsukon et al. [33]. In this study 82% of the 76 study participants agreed that physiotherapists should assess patients’ sleep habits and sleep quality. 95% agreed that patients’ sleep patterns have influenced therapy outcomes. Results demonstrate that most health professionals involved in patient or resident care feel responsible for good sleep and the management of sleep disturbances.

A previous study stated that sleep is not regularly addressed in continuous nursing education [30]. Comparable results were found in this study where 93.2% of the included night nurses reported that they had never received training about sleep and management of sleep disturbances. In Siengsukon et al. [33] nearly 70% of the included participants reported that the majority of physiotherapists did not receive training about sleep after graduation. The lack of training about sleep and sleep management has also been reported in other studies [15]. Also, nursing students reported that they do not feel prepared to manage sleep disturbances after graduation [32]. Therefore, there is a need for continuous nursing education about sleep and its management. Nurses stated to have a moderate level of knowledge about sleep and sleep promotion. Based on the study results it should be mentioned that this knowledge is mainly obtained by self-studying or by experiences with relatives suffering from sleep disturbances, and not through professional training.

As seen in the results of the open-ended questions, where nurses working at night were asked about non-pharmacological interventions to promote sleep, it became clear that they are aware of a variety of non-pharmacological interventions. Similar results were demonstrated in a qualitative study with 13 nurses by Kauffmann et al. (2018), where nurses reported a number of non-pharmacological interventions to avoid older patients’ difficulties in falling asleep during a hospital stay. Authors of this study also identified barriers for the use of non-pharmacological interventions, such as lack of time, shortage of nursing staff, lack of guidelines, and discrepancies between nurses’ attitudes towards the use of non-pharmacological and pharmacological interventions [31]. Most nurses in the present study stated that pharmacological interventions are only used when non-pharmacological interventions are not sufficient, whereby nurses’ perceptions of the effectiveness of non-pharmacological interventions were moderately positive. This underlines that the majority of night nurses act in accordance to guideline recommendations were non-pharmacological interventions are recommended as first intervention of choice to reduce behavior that challenges of people with dementia including sleep disturbances [35, 36]. The results indicate that the use of non-pharmacological instead of pharmacological interventions could be increased by more knowledge on the effectiveness of the two types of interventions. It would be advisable to inform and train nurses with regard to the existing evidence-based recommendations concerning the use of non-pharmacological interventions [17–20], as there is no clear evidence for the effectiveness and safety of pharmacological interventions [16].

Nurses felt unsure if the management of sleep is the responsibility of nurses working at daytime or nurses working at night-time. Although daytime sleepiness was seen as an important problem, nurses felt unclear who is responsible for the management. Therefore, there is a need that nurses are aware of the fact that sleep promotion is a 24-h task, and everyone has to take responsibility. Because up to now, the 24-h responsibility is not implemented. The implementation of designated persons in nursing homes showed improvements in the quality of geriatric care [37]. Therefore, nurses with a special role, such as sleep nurses, could be helpful to increase the 24-h responsibility to monitor the sleep management. Our results indicate the need to improve sleep management in German nursing homes.

Although nurses in this study indicated that sleep is an important topic in nursing homes, the lack of guidelines and the missing documentation of residents’ sleep indicates that sleep is not viewed as an important aspect of the nursing process documentation. In Germany, there are clear guidelines for the nursing process and documentation on numerous topics such as pressure ulcer prevention, mobilization and continence promotion (MDS, 38, MDS, 39). However, there are no guidelines for sleep promotion available. It seems that residents’ sleep is mainly discussed orally during handovers, whereby no standardized procedure is guaranteed due to missing documentation.

Similar results were found in a recent study [30], showing that no policy documents existed in acute-care hospitals. It seems that sleep of residents in nursing homes as well as patients’ sleep in acute-care is not adequately addressed. It is clearly the responsibility of policy makers and nursing managers to face these challenges and to support nurses in order to be able to provide good sleep management.

### Strengths and limitations

This is the first survey exploring attitudes and knowledge regarding sleep and sleep promotion of night nurses and nursing assistants working in nursing homes. The study clearly indicate that night nurses and nursing assistants are an important, and so far insufficiently considered professional group in the context of sleep disturbances. It is a strength of the study that, despite the challenging recruitment of this group, an appropriate sample size of night nurses was reached, compared to other international studies. Nursing homes in Germany are not prepared and have no designated person in charge responsible for research activities. Therefore, the recruitment took place by contacting the nursing home manager or the head nurse. As our recruitment has shown, there is only a small number of nurses performing night shifts and additionally night nurses are difficult to contact because of their special working and rest hours. A further strength is the multicenter design of this survey study, recruiting nurses from different regions in Germany.

Nevertheless, the study has some limitations: First, the response rate (especially in the online survey) was low. Many nursing homes who initially approved to participate in the survey, finally did not take part. The main cause was probable the special burdensome situation for nursing homes and their staff as result of the COVID-19 pandemic. Second, in this context it must also be noted that some nurses included in this study performed a large number of night shifts (more than 20) during the survey period. This could also be a result of the COVID-19 pandemic and the accompanying lack of nursing staff in several nursing homes resulting in a large amount of overtime. It remains unclear what effects this high workload had on the night nurses' attitudes. Third, there was an unbalanced data structure of nurses within nursing homes, ranging from one to fourteen (Median 1) nurses. However, since we do not compare facilities in our analyses, this is negligible. Fourth, the questionnaire was piloted but not validated beforehand. This should be done before further use of the questionnaire. Since night nurses were the target group of our study, it remains unclear whether day nurses share the same attitudes. A further survey would be needed to address the attitudes of nurses only working in day shifts and also including other professions included in care.

## Conclusion

This survey provides information on attitudes and knowledge towards sleep of nurses regularly working in night shifts in German nursing homes. The assessment of residents’ sleep as well as daily documentation and communication regarding sleep is necessary and should be improved. To reach this goal, institutions need to implement guidelines for the management of sleep disturbances in order to develop and implement evidence-based non-pharmacological interventions. In addition, responsibilities for the issue of sleep and sleep problems should be clearly clarified. From our point of view, this is a 24/7 task that can only be solved by a team responsibility in combination with individual champions (e.g. sleep nurses) for the topic.

Results show educational needs of nurses regarding sleep and sleep promotion and underline the development and provision of continuous education. Since nurses have a positive attitude regarding sleep, they would be willing to expand their knowledge about sleep management and take part in training courses. Thus, the development of evidence-based training programs to enable nurses to provide best practice is required.

## Supplementary Information

**Additional file 1.**

## Data Availability

Data is available from the corresponding author on reasonable request.
