# Supplementary material for: Attitudes and knowledge of nurses working at night and sleep promotion in nursing home residents: multicenter cross-sectional survey
Source: BMC Geriatr. 2023 Mar 31;23:206. doi: 10.1186/s12877-023-03928-9 (PMC10066004; doi:10.1186/s12877-023-03928-9)
Supplement: Supplementary file 1 — Additional file 1. [file 12877_2023_3928_MOESM1_ESM.docx]

**Supplement Material**

**Table S1: 56-items questionnaire**

| **Topic** | **No of items** | **Answer option** | **Adapted from** |
| --- | --- | --- | --- |
| 1. Attitudes towards sleep and sleep disturbances | 15 | 5 point Likert Scale plus the additional option “not applicable” | Siengsukon et al. 2015 |
| 1. Importance of sleep in nursing homes | 6 | 4 point Likert Scale  Rating scale from 0 (not important) to 10 (very important) (1 item) | Gellerstedt et al. 2019 |
|  | 7 | Single-answer questions (yes/no/don’t know) |  |
|  | 1 | Open-ended question |  |
|  | 1 | Rating scale from 0 (not important) to 10 (very important) |  |
| 1. Knowledge about sleep and sleep promotion | 5 | Open-ended questions | Kauffmann et al. 2018  McIntosh and MacMillan et al. 2020 |
| 1. Education and training about sleep and sleep promotion | 1 | Single-answer question (yes/no/don’t know) | Wilfling et al. 2020 |
|  | 1 | Rating scale from 0 (no knowledge) to 10 (perfect knowledge) |  |
|  | 1 | Multiple-choice question |  |
| 1. Attitudes towards pharmacological interventions to promote sleep | 11 | 4 point Likert Scale plus the additional option “not applicable” | Kauffmann et al. 2019 |
| 1. Sociodemographic variables | 7 | Demographic questions | Wilfling et al. 2020 |

**Figure S1: Flow Chart**

**Analysis**

**Enrolment**

**773 Nursing homes assessed for eligibility**

**590 Excluded (did not want to participate in the study)**

**183 Nursing homes included**

**45 Nurses participated
in the online survey**

**226 Nurses participated
in the paper and pencil survey**

**138 Nursing homes participated in the survey**

**271 Nurses participated in the survey**

**Table S2: Attitudes towards sleep and sleep disturbances**

| **What I think about residents’ sleep as a professional** | **N** | **Strongly agree** | **Agree** | **Neither** | **Disagree** | **Strongly disagree** | **Unsure** |
| --- | --- | --- | --- | --- | --- | --- | --- |
| Nurses should routinely ask residents about sleep disturbances | 254 | 153 (60.2) | 43 (16.9) | 44 (17.3) | 8 (3.1) | 4 (1.6) | 2 (0.8) |
| Most residents seek medical help for their sleep disorders | 252 | 36 (14.3) | 59 (23.4) | 114 (45.2) | 28 (11.1) | 13 (5.2) | 2 (0.8) |
| Most residents seek nursing help for their sleep disorders | 251 | 31 (12.4) | 63 (25.1) | 114 (45.4) | 29 (11.6) | 9 (3.6) | 5 (2.0) |
| Daytime sleepiness is an important medical symptom | 251 | 105 (41.8) | 61 (24.3) | 57 (22.7) | 9 (3.6) | 13 (5.3) | 6 (2.4) |
| Sleep disorders may contribute to health problems | 252 | 192 (76.2) | 51 (20.2) | 7 (2.8) | 2 (0.8) | 0 | 0 |
| Most sleep disorders are treatable by pharmacological interventions | 251 | 30 (12.0) | 59 (23.6) | 122 (48.6) | 26 (10.4) | 12 (4.8) | 2 (0.8) |
| Most sleep disorders are treatable by non-pharmacological interventions | 251 | 11 (4.4) | 44 (17.5) | 123 (49.0) | 44 (17.5) | 22 (8.8) | 7 (2.8) |
| Sleep disorders are less important than other medical disorders | 252 | 8 (3.2) | 17 (6.7) | 20 (7.9) | 66 (26.2) | 139 (55.2) | 2 (0.8) |
| Most sleep problems are psychiatric | 251 | 27 (10.8) | 64 (25.5) | 113 (45.0) | 14 (5.6) | 31 (12.4) | 2 (0.8) |
| Nurses should ask residents about their sleep habits and sleep quality | 252 | 172 (68.3) | 47 (18.7) | 27 (10.7) | 0 | 5 (2.0) | 1 (0.4) |
| Nurses should perform objective assessments (such as standardized assessment instruments) to assess residents’ sleep habits and sleep quality | 241 | 62 (25.7) | 68 (28.2) | 57 (23.7) | 21 (8.7) | 25 (10.4) | 8 (3.3) |
| Nurses should counsel patients regarding methods to improve sleep quality | 242 | 89 (36.8) | 80 (33.1) | 49 (20.2) | 8 (3.3) | 7 (2.9) | 9 (3.7) |
| Addressing sleep issues may impact nursing care outcomes | 241 | 142 (58.9) | 74 (30.7) | 18 (7.5) | 1 (0.4) | 4 (1.7) | 2 (0.8) |
| Addressing sleep issues is the responsibility of nurses working at daytime | 240 | 28 (11.7) | 18 (7.5) | 100 (41.7) | 27 (11.3) | 57 (23.8) | 10 (4.2) |
| Addressing sleep issues is the responsibility of nurses working at nighttime | 239 | 21 (8.8) | 14 (5.9) | 99 (41.4) | 37 (15.5) | 58 (24.3) | 10 (4.2) |

Results are presented as numbers n and percentages (%). Missing values were pairwise excluded.

**Table S3: Sleep in the nursing process**

| **In the nursing home where I work …** | **N** | **Always** | **Often** | **Seldom** | **Never** |
| --- | --- | --- | --- | --- | --- |
| Residents’ sleep is documented | 238 | 35 (14.7) | 128 (53.8) | 68 (28.6) | 7 (2.9) |
| Documentation regarding residents’ sleep is based on nurse's observations. | 237 | 138 (58.2) | 93 (39.2) | 4 (1.7) | 2 (0.8) |
| Some form of assessment tool is used to assess residents’ sleep | 230 | 13 (5.7) | 22 (9.6) | 47 (21.4) | 148 (64.3) |
| Conventional sleep promotion is applied | 236 | 39 (16.5) | 109 (46.2) | 70 (29.7) | 18 (7.6) |
| Residents’ sleep is a topic discussed on the medical round | 237 | 69 (29.1) | 100 (42.2) | 58 (24.5) | 10 (4.2) |
| Residents’ sleep is a topic that is addressed when reporting/hand over. | 239 | 112 (46.9) | 97 (40.6) | 26 (10.9) | 4 (1.7) |

Results are presented as numbers n and percentages (%). Missing values were pairwise excluded.

**Table S4: Policy documents and care development for sleep**

| **Policy documents and care development for sleep** | **N** | **Yes** | **No** | **Don’t know** |
| --- | --- | --- | --- | --- |
| The nursing home has policy documents providing guidance for nightshifts regarding resident’s sleep | 249 | 54 (21.7) | 119 (47.8) | 76 (30.5) |
| The nursing home has policy documents providing guidance for dayshifts regarding resident’s sleep | 248 | 37 (14.9) | 126 (50.8) | 85 (34.3) |
| Is sleep and residents’ sleep a topic highlighted on further training days? | 250 | 54 (21.6) | 164 (65.6) | 32 (12.8) |
| Does the nursing home have any literature about sleep available? | 247 | 40 (16.2) | 134 (54.3) | 73 (29.6) |
| Is there a designated person in the nursing home responsible for the topic of sleep and residents’ sleep? | 249 | 4 (1.6) | 229 (92.0) | 16 (6.4) |
| Is there a designated person at your ward responsible for the topic of sleep and residents’ sleep? | 250 | 6 (2.4) | 232 (92.8) | 12 (4.8) |
| Have there been any incident reports regarding residents’ sleep in the last year? | 248 | 82 (33.1) | 128 (51.6) | 38 (15.3) |

Results are presented as numbers n and percentages (%). Missing values were pairwise excluded.

**Table S5: Knowledge about sleep and sleep promotion**

| **No** | **Open-ended question** |
| --- | --- |
| **1** | In your opinion, what are the most common causes of sleep problems in nursing home residents? |
| **2** | In your opinion, what are the most important interventions to promote the sleep of nursing home residents? |
| **3** | In your opinion, which skills/competencies are necessary in order to be able to promote the sleep of nursing home residents? |
| **4** | What positive experiences have you already made in dealing with non-pharmacological interventions for sleeping problems? |
| **5** | What negative experiences have you already made in dealing with non-pharmacological interventions for sleeping problems? |

**Table S6: Attitudes towards pharmacological interventions**

| **Attitudes towards pharmacological interventions** | **N** | **Strongly agree** | **Agree** | **Disagree** | **Strongly disagree** | **Unsure** |
| --- | --- | --- | --- | --- | --- | --- |
| Residents’ behaviour is essential for the use of sleep medication | 239 | 102 (42.7) | 95 (39.7) | 19 (7.9) | 12 (5.0) | 11 (4.6) |
| Residents’ personality is essential for the use of sleep medication | 240 | 75 (31.3) | 97 (40.4) | 31 (12.9) | 25 (10.4) | 12 (5.0) |
| The nurse-resident relationship is essential for the use of sleep medication | 235 | 70 (29.8) | 62 (26.4) | 51 (21.7) | 38 (16.2) | 14 (6.0) |
| The physician decides about the application of sleep medication | 237 | 199 (84.0) | 26 (11.0) | 9 (3.8) | 2 (0.8) | 1 (0.4) |
| The nurse decides about the application of sleep medication | 234 | 43 (18.4) | 37 (15.8) | 38 (16.2) | 110 (47.0) | 6 (2.6) |
| We always try to find an alternative to medication | 237 | 81 (34.2) | 92 (38.8) | 41 (17.3) | 15 (6.3) | 8 (3.4) |
| Sleep medication is only used when non-pharmacological interventions are not sufficient | 238 | 110 (46.2) | 78 (32.8) | 28 (11.8) | 12 (5.0) | 10 (4.2) |
| I know the effects and side effects of sleep medication | 240 | 103 (42.9) | 96 (40.0) | 19 (7.9) | 11 (4.6) | 11 (4.6) |
| I feel safe in dealing with sleep medication | 240 | 95 (39.6) | 93 (38.8) | 25 (10.4) | 13 (5.4) | 14 (5.8) |
| Dealing with sleep medication should be improved | 236 | 78 (33.1) | 74 (31.4) | 38 (16.1) | 17 (7.2) | 29 (12.3) |

Results are presented as numbers n and percentages (%). Missing values were pairwise excluded.
